# Thrombosis of the External Jugular Vein: A Rare Complication of a Proximal Humerus Fracture Treated with Collar and Cuff Immobilisation

**DOI:** 10.1155/2014/283790

**Published:** 2014-08-27

**Authors:** Michael Gale, Simon Craxford, Leia Taylor, Helen Montgomery, Simon Pickering

**Affiliations:** Department of Orthopaedics, Royal Derby Hospital, Uttoxeter Road, Derby DE22 3NE, UK

## Abstract

We report the case of an 87-year-old woman who developed a thrombosis of her external jugular vein after sustaining a proximal humerus fracture managed nonoperatively with a collar and cuff. At review in fracture clinic she was found to have an enlarged external jugular vein which was subsequently found to be thrombosed. Her collar and cuff had been applied very tightly and it was felt by the ENT team to be the cause of the thrombosis of her external jugular vein. She was fully anticoagulated with warfarin after subsequently developing a deep vein thrombosis in the subclavian and axillary veins. She made a full recovery following anticoagulation. In this case, we review the potential causes of this rare and underdiagnosed condition, as well as the usual investigations and treatments. We also review the common complications of this fracture and the alternative treatment options available.

## 1. Introduction

Thrombosis of the external jugular vein is a rare and frequently underdiagnosed condition [1]. Cases of thrombosis of the external jugular vein have been attributed to central venous catheterisation [1], head and neck infections, such as in Lemierre's syndrome [1], malignancy [2], intravenous drug abuse [1], and idiopathic [2] and iatrogenic injury [1, 3, 4]. Increasing age, obesity, and associated illness have also been attributed as causes [1, 5]. External compression over the vein has also been reported as a possible cause [1]. We present a case of thrombosis of the external jugular vein in a patient who sustained a proximal humerus fracture and was managed by immobilisation in a collar and cuff. In this case, the thrombosis of the jugular vein was attributed to compression by an overly tight collar and cuff. The patient was initially managed with nonsteroidal anti-inflammatory medications and antibiotics but required anticoagulation with warfarin when she subsequently developed an upper limb deep vein thrombosis.

## 2. Case Presentation

An 87-year-old woman presented to the accident and emergency department after sustaining a fall while on holiday. She complained of pain and reduced range of movement in her left shoulder. Her past medical history included hypertension atrial fibrillation, for which she took aspirin 75 mg once a day. She had previously undergone a thyroidectomy in 2000 for hyperthyroidism. She was otherwise fit and well with no known drug allergies.

**Figure 1:**
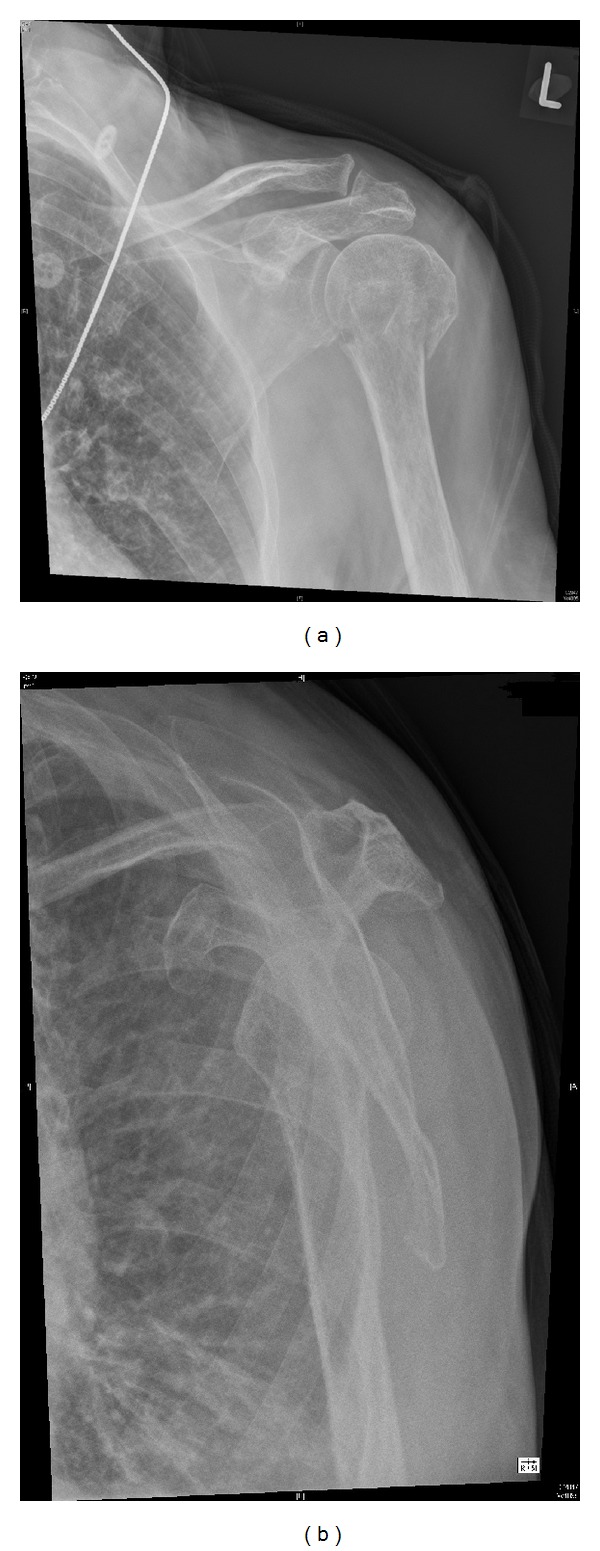
AP and lateral plain film radiographs demonstrating a proximal humerus fracture.

On examination her left arm was bruised and painful when movements were attempted. Her distal neurovascular status of the affected limb was normal with no deficit detected. Plain film radiographs of her left shoulder demonstrated a minimally displaced and impacted fracture of her proximal humerus (as shown in Figure 1). After discussion with the orthopaedic registrar on call she was placed in a collar and cuff and sent home to attend the next available fracture clinic.

**Figure 2:**
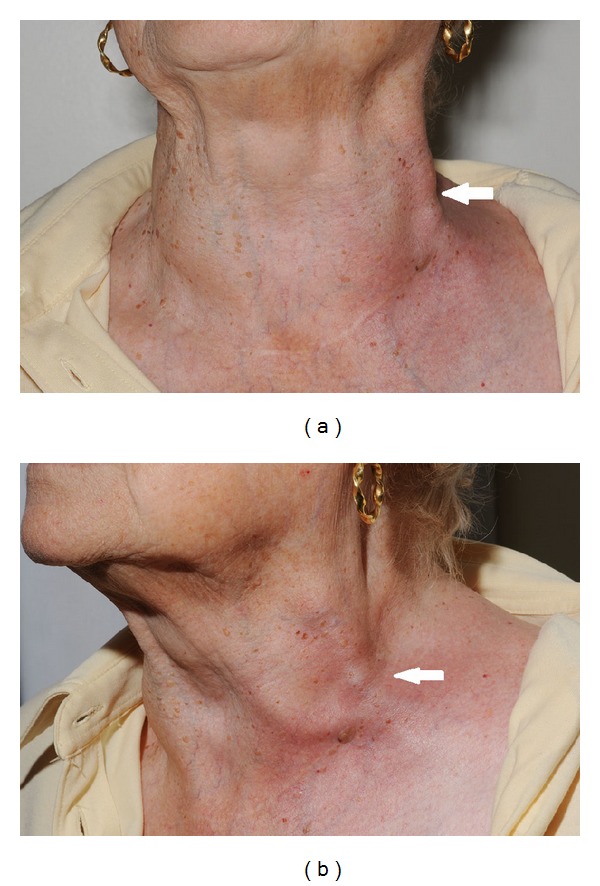
Photographs from fracture clinic demonstrating thrombosis of the external jugular vein with associated superficial phlebitis.

When she was reviewed in the following fracture clinic it was noted that she had developed a swelling and erythema on the left side of her neck in the distribution of her external jugular vein (as shown in Figure 2). It was also found that the collar and cuff had been applied rather tightly. She was referred to the on call ENT registrar who made a diagnosis of thrombophlebitis of her external jugular vein. Further questioning did not reveal a history of further thrombotic events. The ENT registrar felt the erythema may be developing cellulitis and so started the patient on antibiotics (flucloxacillin 500 mg four times a day for 5 days) and placed her in a better fitting sling. The fracture was deemed suitable for nonsurgical management with regular clinical and radiological follow-up. As she was only on holiday in the region, further follow-up was arranged for two weeks in her local hospital.

Two days later, she represented to the accident and emergency department with pain and gross pitting oedema in her left arm. A Doppler scan demonstrated near complete occlusion of the left subclavian and axillary veins consistent with a deep vein thrombosis. She was started on warfarin covered with treatment dose enoxaparin (1 mg/kg once a day) until her INR was therapeutic. As the external jugular vein thrombosis was thought to be triggered by her collar and cuff, the decision was made to anticoagulate the patient for 3 months.

The patient made a full recovery and so far has had no further episodes of thrombosis. While this episode of thrombosis did not significantly impede her rehabilitation, it was a potentially life-threatening complication that perhaps could have been diagnosed earlier in her care.

## 3. Discussion

Fractures of the proximal humerus are common, accounting for around 5% of all fractures [6]. Fracture morphology depends on the mechanism of injury, number of and muscular forces on fracture fragments, and connection of fragments to periosteum [7]. Approximately 75–85% of these fractures show minimal displacement and can be managed nonoperatively [8–11].

Thrombosis of the external jugular vein is a rare and possibly underdiagnosed condition [1]. Previously reported causes include central venous catheterisation [1], head and neck infections, such as in Lemierre's syndrome [1], malignancy [2], aneurysm [12], intravenous drug abuse [1], and idiopathic [2] and iatrogenic injury [1, 3, 4]. Increasing age, obesity, and associated illness have also been attributed as causes [1, 5]. External compression over the vein has also been reported as a possible cause [1]. It appears likely that in our case the thrombosis was caused by compression from a tight collar and cuff.

Clinically thrombosis of the external jugular vein may appear as a swollen, painful elongated mass in the neck [2–4]. There may be associated phlebitis. Further imaging of the external jugular vein includes CT or ultrasound scans; however published data for an imaging protocol is currently lacking [13]. Treatment of a thrombosed jugular vein is controversial and is dependent on the underlying cause. Treatment of an external jugular vein may be different to the treatment of an internal jugular vein thrombosis. Due to the increased risk of further potentially life-threatening thrombotic episodes, such as the risk of pulmonary embolism, patients with a thrombosed internal jugular vein often receive anticoagulation [1, 14]. Some studies have reported that the risk of subsequent pulmonary embolism following thrombosis of the internal jugular vein is around 10% [14]. The risk of deep vein thrombosis associated with external jugular vein thrombosis is less clear; however our case does demonstrate that further thrombosis is possible. While some patients may receive symptomatic treatment with nonsteroidal anti-inflammatory drugs or antibiotics others will require further imaging and anticoagulation, as in our case [13, 15]. It is important that the underlying cause of the thrombosis is identified and addressed. This may require further investigation and an assessment by several different specialities due to the wide range of possible causes for thrombosis.

In conclusion, thrombosis of the external jugular vein is a rare condition that may progress to upper limb deep vein thrombosis. Several causes of thrombosis of the external jugular vein have previously been reported, including external compression as in our case. Treatment options are varied and depend on patient related conditions. The underlying cause should be sought and appropriate follow-up arranged to these patients. While the patient's rehabilitation was not affected and she made an eventual full recovery, an upper limb deep vein thrombosis is a potentially life-threatening condition that could potentially have been diagnosed earlier in our case. It is important to have a low threshold for further investigation in these patients, especially as many patients may present to specialities that have little exposure to the condition.
